# Morphometric Characterization of Hemp Achene and Leaf Trichomes Based on X-Ray Micro-CT

**DOI:** 10.3390/foods15132287

**Published:** 2026-06-25

**Authors:** Laura Gargiulo, Sabrina Maria Marsala, Giacomo Mele

**Affiliations:** 1 Institute for Agricultural and Forest Systems in the Mediterranean (ISAFoM), National Research Council (CNR), P.le Enrico Fermi 1—Loc. Porto del Granatello, 80055 Portici (NA), Italy; sabrinamaria.marsala@unina.it (S.M.M.); giacomo.mele@cnr.it (G.M.); 2 Department of Biology, University of Naples Federico II, Via Vicinale Cupa Cinthia, 21, 80126 Napoli, Italy

**Keywords:** high-resolution plant phenotyping, 3D image analysis, seed morphology, trichomes, *Cannabis sativa* L., morphometric trait

## Abstract

Industrial hemp (*Cannabis sativa* L.) is increasingly being recognized for the production of functional food ingredients and nutraceutical products with broad applications in human nutrition. Its nutrient-rich seeds are of particular interest for their nutritional profile. Moreover, its inflorescences and trichomes provide sources of nutrient-rich proteins, bioactive compounds, and functional substances for food formulations. Agronomic practices, environmental factors, and genotype considerably influence the hemp nutritional profile; thus, continued interdisciplinary research is needed to standardize quality across supply chains. X-ray micro-computed tomography (micro-CT) combined with 3D image analysis is an emerging non-destructive technique in high-resolution plant phenotyping. The aim of this work was to show the contribution of X-ray micro-CT to the quantitative characterization of the internal hemp seed structure and of the trichomes. The 3D image analysis approach used allowed us to determine many morphometric traits of the different seed parts and of the trichomes. Among them, volume ratios of the different seed parts and the density and morphological characteristics of the trichomes of two cultivars were accurately quantified. Overall, this work showed the contribution of X-ray micro-CT in 3D morphometric characterization of the hemp achene structure and trichomes. The obtained seed morphometric traits could be correlated in future applications with nutritional and/or physiological properties of different hemp varieties in order to support different aspects of the whole hemp supply chain such as the dehulling process, oil and protein recovery, seed quality evaluation, and genotype screening, to which trichome characterization could also contribute.

## 1. Introduction

Industrial hemp (*Cannabis sativa* L.) has recently experienced an increase in popularity. The global interest in diversifying its use in various industries, including the food industry, is growing as it is a valuable source of natural ingredients for functional foods [1,2].

Hemp seeds stand out for their remarkable content of quality proteins, including edestin and albumin, two distinct types of proteins that contribute to the seeds’ exceptional nutritional value. Hemp seeds are also rich in healthy lipids with a high content of polyunsaturated fatty acids and some vitamins [1]. For human nutrition, hemp seeds are used in whole form or as flour, or are transformed in order to extract oil [2,3]. Significant differences in oil, protein, and antinutritional component content have also been found between different genotypes [4], suggesting that conventional or molecular breeding may be a potential technique for improving hemp seed quality. Regarding chemical composition, some studies in the literature have highlighted differences in the content of phytocannabinoids or polyphenols between the epicarp of the achene and the real seed (embryo and endosperm) [5]. The chemical profile of *Cannabis sativa* L. varies between genotypes [6] and geographic locations [7]. Moreover, it is affected considerably by cultivation conditions [8] and stress that the plants are exposed to [9].

Besides the relationship between hemp seed parts and chemical component distribution, knowledge of the physical properties of hemp seeds is also essential to facilitate the design of equipment for seed management, harvesting, processing, and storage [10].

In such a framework, 3D imaging of the different achene parts using X-ray micro-computed tomography (micro-CT) could contribute to hemp seed studies. For example, the three-dimensional characterization of the pericarp and of the embryo and endosperm can provide useful information for practical aspects such as the dehulling process and oil and protein recovery, respectively, and also for the evaluation of seed quality or for genotype screening. X-ray microtomography is a non-destructive technique increasingly used to analyze seed quality [11,12]. The studies of Arkhipov et al. [13] and Ma et al. [14] have highlighted that X-ray micro-CT can be recommended for high-resolution seed phenotyping, elucidating many internal seed traits. Morphometric traits of the different seed parts can be correlated with their specific chemical–nutritional data, as shown by Gargiulo et al. [15] for quinoa seeds. The studies of Fang et al. [16] and Datta et al. [17] showed that it is possible to determine the tissue-specific distribution of metabolites in the different seed parts by means of the laser microdissection technique. Such a technique has also been used for the analysis of cannabinoids in different types of trichomes of *Cannabis sativa* L. [18].

Furthermore, as stated by the International Seed Testing Association (ISTA) [19], X-ray micro-CT will be part of standard seed testing in the future. For example, a test based on X-ray microtomography was developed by Porsch [20] for sugar beet seeds. Regarding hemp (*Cannabis sativa* L.), X-ray micro-CT has so far only been used to evaluate the structural characteristics of hemp fibres, analyzed both as natural fibres and in composite materials [21,22,23], but to the best of our knowledge, it is has never been used to characterize hemp seed and trichome structure.

This work aims to demonstrate the contribution of X-ray micro-CT combined with 3D image analysis to the high-resolution morphometric characterization of the internal hemp seed structure and of the trichomes, as both the achenes and trichomes are rich in substances of great nutritional interest.

## 2. Materials and Methods

### 2.1. Plant Materials

Microtomographic analysis was performed on achene, leaf, and stem portions. The achenes were selected from a seed sample of *Cannabis sativa* L. (cv. Felina32) collected when achenes were fully developed from an orchard in Avellino, in the Campania Region in Southern Italy. Regarding the leaves, a comparative analysis was performed on the trichomes of leaves of two different cultivars, Uso 31 and Jubileu, grown in the same experimental field in Caserta, in the Campania Region in Southern Italy. Both the cultivars were sowed in June and grown in dry farming conditions. Approximately 45 days after sowing, i.e., when the two cultivars were at the same developmental stage (flowering phase), samples of well-developed leaves located in the mid-stem area of plants of equal height were taken from both cultivar plants. The three cultivars considered in this work are all monoecious. For X-ray micro-CT scans, one achene and two leaf portions were chosen as representative samples, with the aim of describing the proposed 3D analysis approach and the obtainable morphometric traits. All the analyzed samples were fresh.

### 2.2. X-Ray Microtomography

The X-ray micro-CT scans were performed using the Bruker Skyscan 1272 desktop microtomograph (Bruker, Kontich, Belgium). The system is equipped with a cone beam X-ray source adjustable in the 20–100 kV energy range and allows a cylinder-shaped volume of 6.5 cm in diameter and 7.2 cm height as the maximum sample size. Preliminary tests were conducted to identify the optimal microtomographic scanning parameters for both the achene and the leaf portions to obtain satisfactory results in terms of image quality. The aim was to obtain an accurate morphometric analysis of the different achene parts and of the trichomes.

Achene image acquisition was performed at a source voltage and current of 50 kV and 200 μA, respectively. The X-ray scans were acquired at a resolution of 6.6 μm voxel size. The distance between the achene and the X-ray source was 40.4 mm. The rotation step was set to 0.4 degrees, and the frame averaging was set to 10 to reduce image noise. The X-ray projection acquisition process required approximately 1 h and 10 min to obtain 471 two-dimensional X-ray projection images for the scan.

For the acquisition of the leaf tissue portions, source voltage and current were set at 33 kV and 300 μA, respectively. The X-ray source/sample distance was 65.5 mm, and the rotation step was set to 0.2 degrees. The X-ray scans were acquired at a resolution of 3 μm voxel size. The X-ray projection acquisition process required approximately 2 h and 10 min to obtain 800 two-dimensional X-ray projection images for the scan.

### 2.3. 3D Image Processing

X-ray projection images were used to reconstruct 3D images using the NRecon software, version 2.2.0.6 (Bruker, Kontich, Belgium). The reconstruction procedure comprised a filtered back projection algorithm [24]. Ring artefact and smoothing corrections were applied at 20% and 5% levels, respectively, to obtain a proper 3D reconstruction of the images.

The cross-section images were binarized using Otsu’s thresholding method [25] to segment the different achene parts with different mean grey values and isolating the separated achene parts if they showed the same grey level. This twofold approach allowed for a reliable segmentation of the embryo, the endosperm, and the pericarp. Image binarization was performed using the CTAn software, version 1.23.0. (Bruker, Kontich, Belgium).

### 2.4. 3D Image Analysis

Morphometric traits of the whole achene and of the different achene parts were measured by applying both the so-called “Object-based Image Analysis” (OBIA) and the mathematical morphology approaches, described, for example, by Blaschke et al. [26] and Dougherty et al. [27], respectively.

The OBIA method allowed the quantitative characterization of the morphology of the whole achene and of its different parts as isolated 3D objects. They were measured using the software Image ProPremier 3D, version 9.2 (Media Cybernetics, Rockville, MD, USA).

For the characterization of pericarp structure, its thickness distribution and the derived average pericarp thickness were determined using the mathematical morphology algorithm of ‘successive opening’ with spherical ‘structuring elements’, described by Dougherty et al. [27]. These parameters were determined using the software CTAn, version 1.23.0.2 (Bruker, Kontich, Belgium).

All the morphometric parameters determined for each achene part are described in Table 1.

The OBIA approach was applied also to determine the diameter and the height of all the segmented trichomes. The number of trichomes was automatically counted from the X-ray projection images of leaf tissue portion using the software Image ProPremier 3D, version 9.2 (Media Cybernetics, Rockville, MD, USA).

## 3. Results

### 3.1. Achene

**Figure 1:**
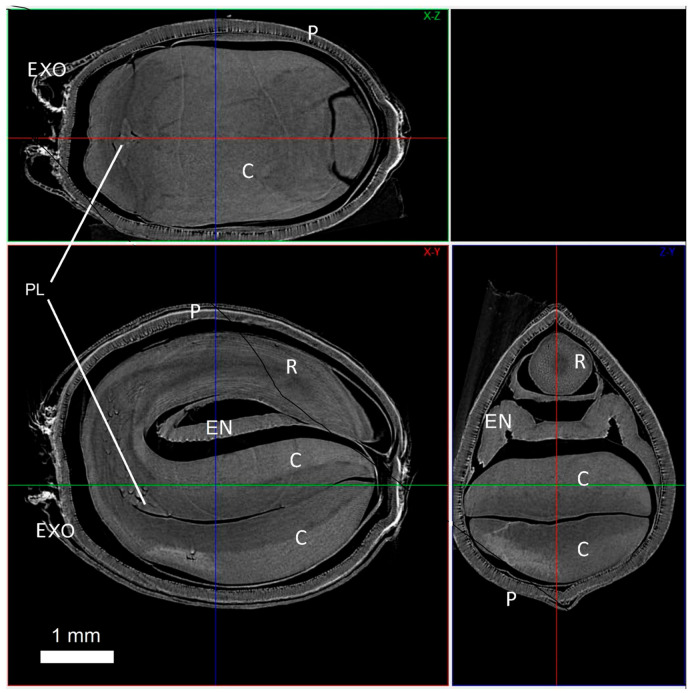
Achene parts. Hemp (cv Felina32) achene cross-sections showing the different internal parts: exocarp (EXO), pericarp (P), cotyledons (C), radicle (R), endosperm (EN), plumule (PL). The coloured lines indicate the tranveral (red), sagittal (green), coronal sections correponding to the showed transversal (X-Y), sagittal (X-Z), coronal (Z-Y) planes.

Microtomographic analysis of the achene allowed us to obtain a detailed three-dimensional reconstruction of its internal anatomy. Figure 1 shows three cross-sections in which the achene’s internal components are clearly distinguishable.

**Figure 2:**
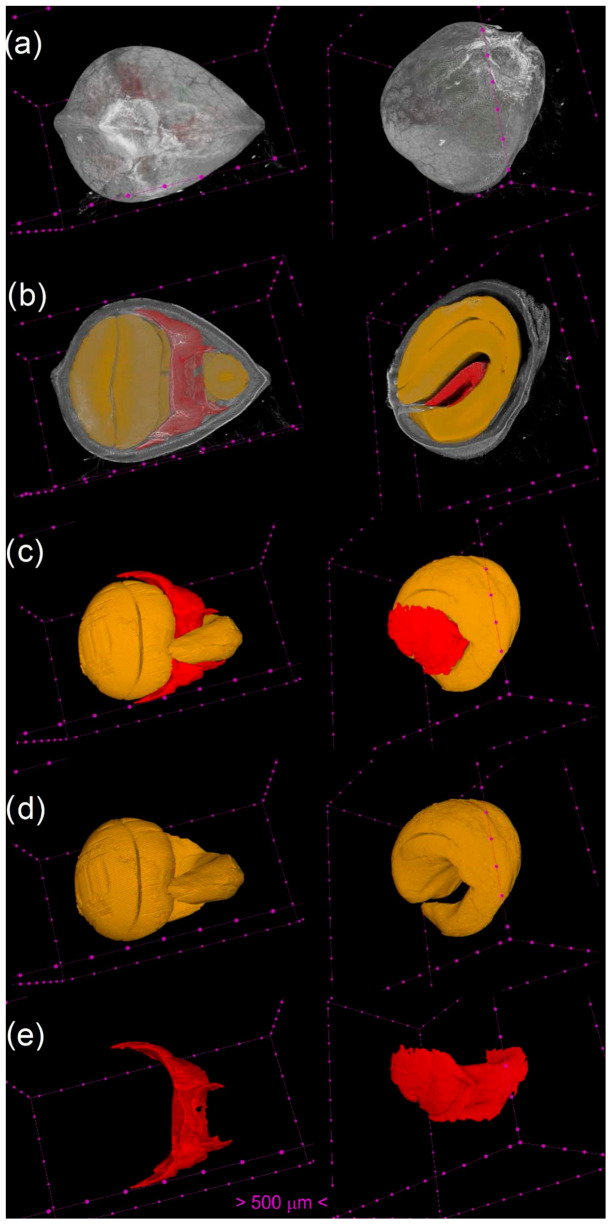
Double view of three-dimensional reconstructions of the hemp achene (cv Felina32) and its parts. (**a**) External view of the achene; (**b**) achene section with embryo (yellow) and endosperm (red); (**c**) the real seed; (**d**) the embryo; (**e**) the endosperm. The distance between the pink dots of the virtual boxes containing each 3D reconstruction is 500 microns.

**Figure 3:**
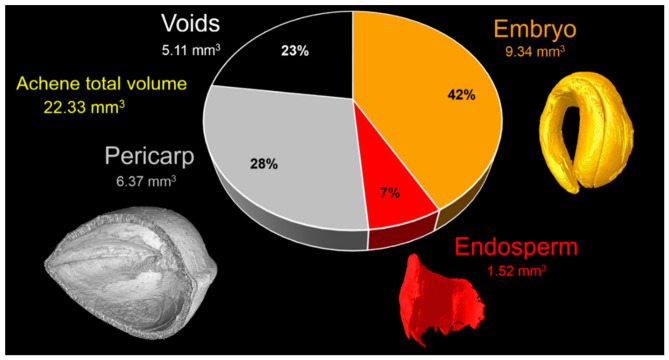
Pie chart showing the volumes as percentages of the achene parts and the three-dimensional reconstructions of the analyzed parts with their volumes in mm^3^. The data refer to a single representative achene.

The identification of the different parts of the achene from the 3D images allowed us to identify, isolate, and characterize the three-dimensional structure of the pericarp, embryo, and endosperm, as can be observed from the images shown in Figure 2 and from Supplementary Video S1. The three-dimensional reconstruction allows us to evaluate the reciprocal spatial relationships between the components, while the quantitative morphometric image analysis allows us to determine their volumetric relationships (Figure 3).

As can be seen from the pie chart in Figure 3, the volumes of the embryo and endosperm together represent approximately half of the volume of the whole achene, almost entirely occupied by the embryo, while the other half of the volume is almost equally divided between the pericarp and the cavity inside the achene.

A morphometric characterization of the whole achene and its internal components was also performed as a representative example of the obtainable morphometric traits, and the results are shown in Table 2. Although the achene’s sphericity value is very close to 1, the Feret diameter values and their ratios show that its shape is almost ellipsoidal. Furthermore, the embryo, despite having a volume equal to less than half of the entire achene, has a surface area almost equal to that of the external surface area of the pericarp.

**Figure 4:**
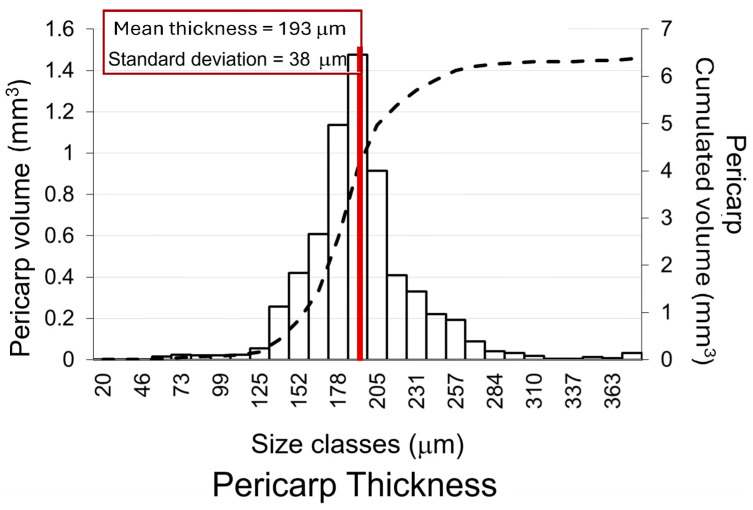
Pericarp thickness distribution. The horizontal axis reports the pericarp thickness size classes in microns, while the primary vertical axis (on the left) reports the pericarp volume of each thickness class, and the secondary axis reports the cumulated volume of the pericarp thickness classes. Dashed line indicates the cumulated thickness distribution.

Regarding the pericarp, the quantity distribution of pericarp thickness and its mean thickness were determined by means of a 3D image analysis approach based on mathematical morphology algorithm of ‘successive opening’ with spherical ‘structuring elements’, described by Dougherty et al. [27]. As can be observed from the three-dimensional reconstruction of the pericarp in Figure 3, Supplementary Video S2, and the histogram shown in Figure 4, the pericarp has low thickness variability (leptokurtic quantity distribution), with most of the volume showing a thickness very close to the mean value of 193 μm.

### 3.2. Leaves and Trichomes

**Figure 5:**
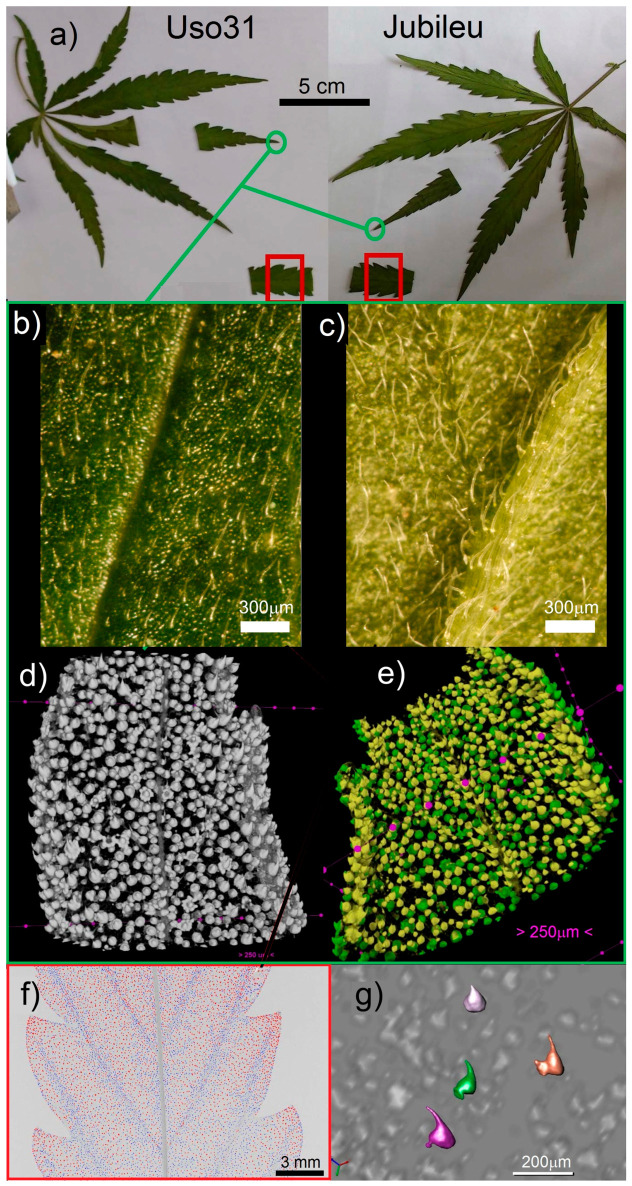
(**a**) Sampled hemp leaves and analyzed leaf portions. Circled in green are the portions used for the optical microscopy image acquisition and 3D morphometric characterization of the trichomes. The red boxes show the portions of leaf used to calculate trichome number. (**b**,**c**) Optical microscopy fields (40×) under visible incident light of adaxial and abaxial surfaces of the leaf. (**d**) 3D reconstruction of the leaf portion with the trichomes. (**e**) 3D reconstruction of the trichomes on the adaxial (green) and abaxial (yellow) surfaces of the leaf. (**f**) X-ray projection image of the leaf area with the automatically counted trichomes, showed as dots. (**g**) 3D reconstruction of some individual non-glandular trichomes.

In Figure 5 are reported the hemp leaf portions analyzed by means of X-ray micro-CT. The smaller portions (green circles in Figure 5a) have been scanned, and their 3D reconstructions are shown in Figure 5b,c as well as in Supplementary Video S3. The higher X-ray attenuation coefficient of the trichomes compared to the leaf tissue allowed for the segmentation of the non-glandular trichomes from the leaf tissue, as shown in Figure 5b. The 3D OBIA approach allowed us to identify the trichomes of the adaxial and abaxial surface of the leaf portions (Figure 5c) and their morphometric characterization is reported in Table 3. The results of the morphometric characterization of the trichomes highlighted a slightly higher size of the leaf trichomes for the “Uso31” cultivar compared to the “Jubileu” cultivar, with a slightly higher density on the abaxial surface of the leaf compared to the adaxial surface for both cultivars.

Regarding the determination of trichome density, the automatic counting of trichomes from X-ray projection images (Figure 5d) allowed us to obtain the total number of trichomes present on both surfaces of the analyzed leaf portion. From the three-dimensional reconstructions of the smaller leaf portions (circled in green in Figure 5a and reported in Figure 5b,c), the trichomes present on the abaxial and adaxial surfaces were separately segmented and counted. The obtained ratio between the number of trichomes present on the two different surfaces was used to calculate the number and therefore the density of trichomes present on the two surfaces of the largest leaf area (shown in red in Figure 5a and reported in Figure 5d).

From the results reported in Table 3, it can be noted that for the cultivar Uso31, the number of trichomes was approximately the same on the two different surfaces, while in the cultivar Jubileu, the trichome number, and obviously the density, was higher on the abaxial than on the adaxial surface.

## 4. Discussion

The obtained results represent an example of the contribution of X-ray micro-CT combined with 3D image analysis to the high-resolution morphological phenotyping of hemp seeds and trichomes. The obtained morphological traits of seed components may indeed be very useful for the characterization of hemp seeds as a food or nutraceutical feed. They have the potential to be correlated with nutritional and/or physiological properties. In future work, for example, the volumes or the volume ratios of the different achene parts could be related to the content of different substances, such as fatty acids, tocopherols, and antinutritional components, as well as concentrations of crude protein and oil, combining the proposed approaches to chemical analysis and contributing to the characterization and selection of seeds of different hemp genotypes [3,28].

The 3D morphometric seed traits identified on a representative sample in this work can be effectively determined on a statistically significant number of achenes of different genotypes. In this way, they have the potential to be used in the future in advanced multivariate statistical analysis procedures with the aim of contributing to the seed sorting or breeding of different hemp genotypes, improving the sustainability of the hemp seed supply chain. X-ray micro-CT is indeed being increasingly used for non-destructive and high-throughput phenotyping of seeds [29].

The OBIA approach, although applied in this study only to the non-glandular trichomes of the leaf as an example, can also be effectively used to morphometrically characterize other types of trichomes present on the hemp plant, particularly the pedunculated ones, typical of the inflorescence, and the sessile and bulbous ones present in other parts of the plant [30]. The proposed approach for the quantification of the trichome density using the X-ray projections allows for the automatic counting of trichomes on a significant number of leaf or bract portions, contributing to the characterization of the spatial distribution of the hemp glandular trichomes [30]. Combining such morphometric characterization with chemical analysis of microdissected trichomes [18] could allow for the trichome density to be related to metabolite concentrations.

Overall, this work aimed to describe in detail the proposed methodological approach. As representative examples, 3D morphometric characterization was performed only on one achene and a limited number of trichomes, not yet combining the obtained results with chemical analysis or statistically comparing the obtained results.

Notwithstanding such limitations, the obtained results provide high detail and accuracy for the quantitative anatomical characterization of not only hemp achene but also trichomes, opening new possibilities for the phenotypic characterization of this species of considerable agronomic and nutritional interest.

## Figures and Tables

**Table 1:** Morphometric parameters determined using image analysis, including their definitions and measurement units.

Parameter	Description	Unit
Area of surface	Surface area of object	mm^2^
Volume	Volume of object	mm^3^
Diameter	Equivalent diameter of the object	mm
Sphericity	$\sqrt[{3}]{\pi {\left(6\cdot Volume\right)}^{2}}/Area\;of\;Surface$ For a spherical object this parameter equals 1; for all other shapes it is less than 1	-
Feret max	Maximum distance between two parallel plans enclosing object	mm
Feret min	Minimum distance between two parallel plans enclosing object	mm
Feret ratio	Ratio between Feret min e Feret max	-

**Table 2:** Morphometric parameters of the whole achene chosen as a representative example (cv. “Felina32”) and its parts determined by means of OBIA approach.

Morphometric Parameters	Achene	Embryo	Endosperm	Pericarp
Volume (mm^3^)	22.33	9.34	1.52	6.37
Surface area (mm^2^)	42.80	39.60	20.52	42.80
Equivalent diameter (mm)	3.49	2.61	1.43	3.49
Sphericity	0.90	0.54	0.31	0.90
Feret max (mm)	4.88	3.78	3.00	4.88
Feret min (mm)	2.80	2.28	1.78	2.80
Feret ratio	1.74	1.66	1.69	1.74

**Table 3:** Comparison of morphometric traits of trichomes on the adaxial and abaxial surface of the leaf portion for each cultivar: Uso 31 and Jubileu.

Morphometric Traits	cv. Uso31			cv. Jubileu		
	Adaxial Surface	Abaxial Surface	Total	Adaxial Surface	Abaxial Surface	Total
Leaf area (cm^2^)		2.26			2.37	
Trichome number (NT)	14,466	14,898	29,364	13,734	14,788	28,522
Trichome density (NT/mm^2^)	64.0	65.9	129.9	57.9	62.4	120.3
Mean diameter (μm)	68.5	57.1	62.8	66.3	55.3	60.8
Mean volume (µm^3^)	196,980	110,898	153,939	178,902	100,720	139,695
Mean height (μm)	94.2	82.7	88.4	89.3	77.8	83.6
NT adaxial/NT abaxial	0.97			0.93		

## Data Availability

The original contributions presented in this study are included in the article/Supplementary Material. Further requests can be directed to the corresponding author.

## Supplementary Materials

The following supporting information can be downloaded at https://www.mdpi.com/article/10.3390/foods15132287/s1: Video S1: 3D reconstruction of the hemp achene (cv. Felina32) and its different parts (pericarp in grey, embryo in yellow, endosperm in red). Video S2: 3D reconstruction of the pericarp (cv. Felina32). Video S3: 3D reconstruction of the leaf tissue portion with the segmented trichomes on the adaxial (in green) and abaxial (in yellow) surfaces of the leaf.
